# Short stature in children of Karapotó ethnic background, São Sebastião, Alagoas, Brazil

**DOI:** 10.1016/j.rppede.2016.02.006

**Published:** 2016

**Authors:** Samara Bomfim Gomes Campos, Risia Cristina Egito de Menezes, Maria Alice Araújo Oliveira, Danielle Alice Vieira da Silva, Giovana Longo-Silva, Juliana Souza Oliveira, Leiko Asakura, Emília Chagas Costa, Vanessa Sá Leal

**Affiliations:** a Universidade Federal de Alagoas (UfAl), Maceió, AL, Brazil; b Universidade Federal de Pernambuco (UFPE), Vitória de Santo Antão, PE, Brazil

**Keywords:** Indigenous population, Nutritional status, Anthropometry, Malnutrition

## Abstract

**Objective:**

To describe the prevalence of short stature among children of Karapotó ethnic background.

**Methods:**

Cross-sectional, population-based study that included children between 6 and 59 months of age from the Plak-Ô native village and the Terra Nova settlement, São Sebastião, Alagoas, carried out between 2008 and 2009. Short stature was evaluated by the Height/Ageindex, using as cutoff z score≤−2. The prevalence of short stature was determined by compa-ring simple and relative frequencies. The population growth curves were compared to the WHO reference curves. Data analysis included the outcome variable: Height/Age and the predictor variables: place of residence, gender, age, anemia, birth weight, family income, maternal literacy. The chi-square test was used to compare the categorical variables, where as the chi-square test with Yates correction was used for dichotomous variables, considering as statistically significant *p*-values≤0.05.

**Results:**

The prevalence of short stature was 15.6% for children from the Terra Nova settlement and 9.1% for those from Plak-Ô native village. The prevalence of short stature among the Karapotó ethnicity was 13.4%. The variables: maternal literacy, family income and low birth weight were statistically associated with short stature.

**Conclusions:**

The observed short stature prevalence rates are significant, being characterized as a public health problem. Among the associated factors, the following are noteworthy: unfavorable conditions of maternal literacy, family income and low birth weight. The planning of strategies to reverse the situation must take such factors into consideration.

## Resumo

**Objetivo:**

Descrever a prevalência do déficit estatural entre crianças da povo karapotó.

**Métodos:**

Estudo transversal, de base populacional, incluiu crianças entre seis e 59 meses da aldeia Plak-Ô e do povoado Terra Nova, São Sebastião (AL), feito entre 2008 e 2009. O déficit estatural foi avaliado pelo índice estatura/idade, adotou-se como ponto de corte escore z≤−2. A prevalência de déficit estatural foi determinada pela comparação das frequências simples e relativas. As curvas de crescimento da população foram comparadas com as curvas de referência da OMS. A análise dos dados contemplou a variável de desfecho estatura/idade e as variáveis preditoras local de moradia, sexo, idade, anemia, peso ao nascer, renda familiar, alfabetização materna. Para comparação das variáveis categóricas dos grupos foi usado o teste do qui-quadrado e o teste do qui-quadrado com correção de Yates para as variáveis dicotômicas, consideraram-se como significância estatística *p*-valores≤0,05.

**Resultados:**

A prevalência de déficit estatural foi de 15,6% para as crianças do povoado Terra-Nova e 9,1% para as da aldeia Plak-Ô. A prevalência de déficit estatural da povo karapotó foi de 13,4%. As variáveis: alfabetização materna, renda familiar e baixo peso ao nascer se relacionaram estatisticamente com o déficit estatural.

**Conclusões:**

As prevalências de déficit estatural verificadas são expressivas, são caracterizadas como problema de saúde pública. Destacam-se, entre os fatores associados, condições desfavoráveis de alfabetização materna, renda familiar e baixo peso ao nascer. O planejamento de estratégias para mudar a situação precisa levar tais fatores em consideração.

## Introduction

Despite their importance, there are few available studies on the social, demographic and epidemiological profile of the Brazilian indigenous population, especially the food and nutritional profile. In spite of this lack of information, recent studies show a social marginalization, which has resulted in negative impacts on the health-disease process of these populations.^1^^-^ 3 Regarding the nutritional profile of the indigenous populations in Brazil, it is clear that they are going through a complex transition process, characterized by the increase in overweight among adults and the persistence of health problems related to nutritional deficiencies, especially delayed growth in children.^4^^-^ 6 This situation can be attributed to considerable social diversity involved, if one considers that Brazil has recorded more than 200 indigenous populations throughout the national territory.^7^ As for the delayed growth of indigenous children, there are records of populations in which more than half was identified as being affected by the disorder. This scenario could have serious implications for the health of this population.^8^

Thus, for Brazil's indigenous population, there is an important overlap of diseases: the increase in overweight among adults, whereas the occurrence of malnutrition in children has not been solved.^1^^,^ 7 ^,^ 9 Considering these issues have not yet been fully explored and in order to contribute to a better understanding of the disease dynamics in this population, this study aimed to describe the prevalence of stunting among children of *karapotó* ethnic background residing in two locations of the State of Alagoas, with the purpose of assessing the disease during a period of rapid nutritional transition the country has been going through.

## Method

These data are part of the research ‘Food and nutrition surveillance: implementation of sentinel area in two populations of *karapotó* ethnic background'. This is a cross-sectional study, which considered as study unit the population of children between six and 59 months living in the Plak-Ô reservation and Terra Nova settlement, in São Sebastião, state of Alagoas. The procedures employed in the survey, regarding the study population and data collection, have been previously described.^9^

The modeling process was performed by census survey carried out by native health agents, who identified the children whose age was within the age range specified by the study. Of these, five children were not found at the time of data collection, so the study population consisted of 98 children. One child was excluded due to anthropometric data inconsistency and the final sample consisted of 97 children.

The fieldwork was carried out between 2008 and 2009 by appropriately trained team, who went to the reservation and the settlement four times for data collection.

Interviews were carried out with mothers/tutors of the children through home visits and questionnaires, consisting of questions related to socioeconomic, demographic and biological information. After the questionnaires were filled out, they were reviewed to assess and correct inconsistencies about the collected information.

The collection of anthropometric data and hemoglobin measurements were carried out at the reservation and the settlement. The anthropometric assessment was performed after the team's training, according to the recommendations of the World Health Organization (WHO) and the Brazilian Ministry of Health.^10^^,^ 11 Body weight was obtained using an anthropometric scale, with a capacity of 200kg and precision of 50g. To measure the length of children younger than 24 months, a wooden infantometer was used, with a range of 100cm and precision of 0cm, and for older children, a vertical stadiometer was used, with a range of 213cm. These children were weighed and measured barefoot with minimal clothing. Children aged up to two years were measured in the supine position and those between two and five years old in the standing position. Two measurements were collected and their corresponding means were calculated.

The Anthro 2007 software was used to evaluate the nutritional status, using the height/age index for the diagnosis of stunting/malnutrition. The WHO growth curves were used as a reference.^12^^,^ 13 The classification of children according to height/age was expressed in *z*-scores, using the cutoff points for stunting (≤−2*z* scores) and normal height (>−2*z* scores).

For the diagnosis of anemia, hemoglobin (Hb) was measured using a Hemocue portable hemoglobin analyzer (Fresenius Kabi, Uppsala, Sweden), following the standard procedure of collecting a blood drop by fingertip puncture. The cutoff used for anemia diagnosis was hemoglobin <11g/dL.^14^ Iron supplementation was provided to all children diagnosed with anemia, with the support of the Special Indigenous District (DSEI-AL).

For the stool sample collection for parasitological analysis, containers were handed to the parents/tutors, who were asked to collect stool in their respective households. The fecal samples were collected, placed in a refrigerated cool box and sent for laboratory analysis using the spontaneous sedimentation and Kato-Katz methods.^15^^,^ 16

Double data entry, as well as validation and processing of the analysis were carried out using the Epi-Info software, version 6.04 (CDC, Atlanta, USA). The analyzed variables were categorized as place of residence: Plak-Ô reservation and Terra Nova settlement; garbage collection: classified as ‘adequate' (garbage collected) or ‘inadequate' (garbage was buried, burned or placed in a vacant lot). The variable child's birth place was divided between those who were born at the hospital or maternity and those who were born at home. About vitamin A supplementation, the child's health card was checked regarding the micronutrient supplementation record. Children born weighing ≥2500g were classified as having ‘adequate weight'. Those born weighing <2500g were classified as having ‘inadequate weight', with the information being obtained from the child's health card.^17^ The per capita family income (income earned in the month preceding the interview) was classified as lower and higher than a minimum wage. The maternal level of education was classified as mothers who could read and write and illiterate ones.

The prevalence of stunting was determined by comparing the simple and relative frequencies. To compare categorical variables, we used the chi-square test or chi-square test with Yates correction and the value of *p*≤0,050 was considered as statistically significant for dichotomous variables. To compare the growth curves of the studied population with the WHO reference curves, charts for height/age were prepared using the Anthro software, according to the place of residence.^12^ The project was approved by the Institutional Review Board, under protocol number 009 429/2006-15. The study was submitted to and approved by the National Research Ethics Commission, as required by the decree on studies of indigenous populations, under protocol number 1,013,414.

## Results

The characterization of the population is described in Table 1, which showed a predominance of the female gender (54.6%) and those aged ≥2 years (67%). It also shows that 66% of the children from the studied population live in the Plak-Ô reservation and 34% in the Terra Nova settlement.

**Table 1:** Characterization of the population of children younger than five years of the *karapotó* people. São Sebastião-Alagoas, 2008/2009.

Variables	*n* with information	%
*Place of residence*		
Plak-Ô	33	66.0
Terra Nova	64	34.0

*Gender*		
Male	44	45.4
Female	53	54.6

*Age*		
<2 years	32	33.0
≥2 years	65	67.0

*Garbage collection*		
Adequate	-	-
Inadequate	97	100.0

*Birthplace* a		
Maternity/hospital	94	97.9
Home	2	2.1

*Vitamin A supplementation* a		
Yes	67	77.0
No	20	23.0

*Iron deficiency anemia*		
No	41	42.3
Yes	56	57.7

*Presence of parasitic infestation* a		
No	24	32.9
Yes	49	67.1

a Sample loss as described.

**Figure 1:**
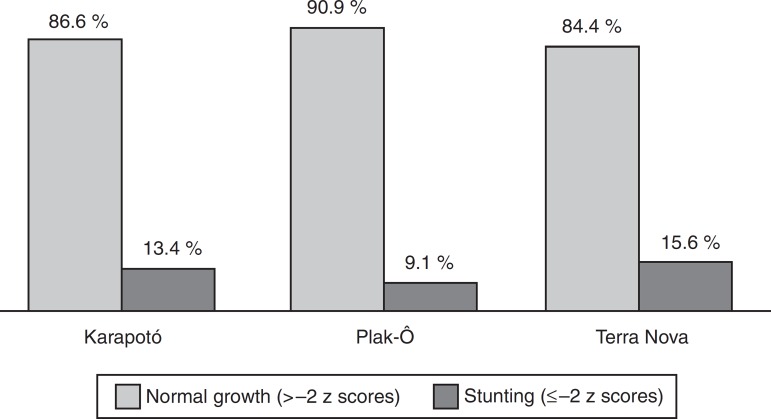
Nutritional status according to height/age index in children younger than five years of the *karapotó* people, by place of residence. São Sebastião-Alagoas, 2008/2009.

A prevalence of stunting of 15.6% was observed in children whose families lived in the Terra Nova settlement and 9.1% for those living in the Plak-Ô reservation. For children of *karapotó* ethnicity, the prevalence of stunting was found to be 13% (Fig. 1). The stunting, in mean *z*-scores, was not significantly associated with gender, age, presence of anemia and place of residence. For the variables maternal education, family income and birth weight, statistically significant differences were found among the analyzed categories and we observed lower mean *z* scores for height/age among children with low birth weight, those whose mothers were illiterate and in those belonging to families with a per capita income <one minimum wage (Table 2).

**Table 2:** Stunting, in mean *z* score, in children younger than five years of *karapotó* people, according to child and maternal variables. São Sebastião-Alagoas, 2008/2009.

Variables	*n*	%	Height/age *z* score	
			x±DP	*p*-value
*Place of residence*				
Plak-Ô	33	66.0	-0.27±1.52	0.288
Terra Nova	64	34.0	-0.68±1.24	

*Gender*				
Male	44	45.4	-0.43±1.33	0.228
Female	53	54.6	-0.64±1.37	

*Age (years)*				
<2	32	33.0	-0.56±1.32	0.894
≥2	65	67.0	-0.54±1.37	

*Anemia in children*				
No	41	42.3	-0.29±1.32	0.098
Yes	56	57.7	-0.73±1.35	

*Birthweight*				
Adequate	88	90.7	-0.46±1.28	0.025
Inadequate	9	9.3	-1.35±1.80	

*Family income* a				
≥1 minimum wage	56	58.3	-0.22±1.40	0.026
<1 minimum wage	40	41.7	-0.78±1.28	

*Maternal literacy*				
Can read and write	62	63.9	-0.30±1.25	0.011
Illiterate	35	36.8	-0.97±1.42	

a Value for the minimum wage in 2008/2009.

**Figure 2:**
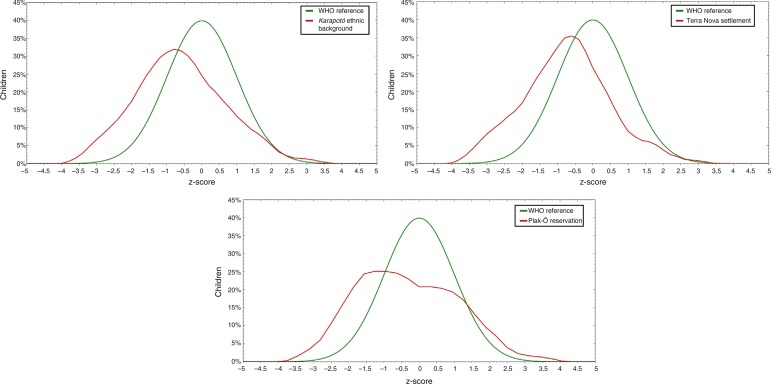
Comparison of the growth curve of children younger than five years old of the *karapotó* people, with the WHO curve, according to height/age index, by place of residence. São Sebastião-Alagoas, 2008/2009.

Fig. 2 shows a comparison of growth deficit according to the height/age index of the studied population, with the WHO reference curves. The children showed, in general, lower values of *z*-score when compared to the analyzed references points, and showed, in the two places of residence, growth curves with a left-trend slope.

## Discussion

The assessed population inhabits two locations in a city in northeastern Brazil, Sao Sebastião, state of Alagoas, a semiarid region. The *karapotó* people consists of an estimated population of 1067 inhabitants, whose lifestyle and means of support resembles those of the non-indigenous population of Brazil.^18^

Regarding the environmental condition, particularly regarding the garbage collection variable, it is observed that the assessed population did not have access to adequate collection of waste during the analyzed period. This condition, as well as the high rate of intestinal parasitic infestation (67%), among other issues, has often been associated with malnutrition in children.^1^^,^ 19 In the context of multiple causes associated to the disease, these sanitary conditions should be considered to establish the nutritional care, which is usually a long process, especially when the implementation of measures to improve environmental sanitation and quality of life has not been planned.^1^^,^ 2

In spite of access to hospital childbirth identified in almost all the studied population (approximately 98%), poor access of these people to health services is shown by the low coverage of supplementation with vitamin A megadoses and it is noteworthy that 23% of the population did not receive this supplementation. Malnourished children often had vitamin A deficiency, which surely compromises their prognostic.^20^ Vitamin A should be administered at prophylactic doses, as determined by the Brazilian strategy of disease control.^21^ The administration of this vitamin would provide these children with increased resistance to infections, particularly in the respiratory and digestive system, and would prevent acute respiratory infections and diarrhea, extremely common diseases at this stage of life.^21^

Another nutritional problem identified in this population was iron-deficiency anemia, which affected almost 60% of the population in this period. This type of anemia, common among Brazilian children, negatively affects the individual's neurological development and cognitive capacity, especially when it occurs early (in the first years of life), the most vulnerable age group to the disease.^22^

Stunting affects more than 13% of children, a higher prevalence than the one found among non-indigenous children in the state of Alagoas (10.3%).^23^ It is noteworthy that the prevalence found here is almost twice that of non-indigenous children in Brazil (7%), according to the National Demographic and Health Survey.^24^

Important reports of malnutrition among indigenous children were recorded in the National Survey of Indigenous People's Health and Nutrition/2009.^7^ The study brought to light relevant information on the profile of indigenous people in Brazil, as it shows the presence of nutritional transition in this population, with the emergence of overweight (30.2%) and obesity (15.7%) in women aged 14-49 years, whereas the occurrence of malnutrition in children has not been solved. The report discloses that 26% of the children younger than five years old have stunting, with even higher rates in the northern region of Brazil (41.1%).^7^

Regarding delayed growth according to the children's place of residence, gender and age, there were no significant differences from a statistical point of view. This fact indicates that regarding the assessed categories, the disease affected children, in terms of magnitude, to the same extent.

Using the −2 standard deviations as a limit implies that 2.3% of the reference population is classified as ‘malnourished', even if the individuals are actually ‘healthy' and have no growth difficulties. Therefore, 2.3% can be considered as the ‘baseline value' or expected prevalence. Although the prevalence rates in disadvantaged populations are generally higher than 2.3%,^25^ the significant nutritional vulnerability of this population is demonstrated by the values found herein (approximately 9-16%, according to the place of residence), being four to seven-fold higher than the threshold value. Although it is clear that Brazilian indigenous children still show a high prevalence of delayed growth, the understanding of the main factors related to this disease is still limited.^7^^,^ 8

Regarding the association between stunting and children's age, the results shown here are similar to the studies with the *Kaingang* children, for which age was not significantly associated with weight deficit.^26^ In contrast, a study carried out with the xavante people showed an inverse association between stunting with the child's age: the younger the age, the better the nutritional state.^27^ The study authors pointed to breastfeeding as a protective factor for the disease in the studied group.

Maternal literacy favorably influenced the nutritional status of their children. Higher mean *z* scores were identified among the children of literate mothers. When these findings are compared with studies carried out in the general population, the results shown here corroborate the literature. Studies indicate low maternal schooling as a predictive factor for malnutrition in children. Unquestionably, malnutrition is more common in individuals with low educational levels, due in part to the association between maternal education and inclusion of women in the labor market, which allows the increase in family income and directly affects food availability and access.^28^^,^ 29

Regarding the analysis of the families' income, it is known that involves different aspects that hinder the interpretation of these data, as it is considered that traditional communities, such as those of indigenous population, constitute social organizations that operate economical models with little capital accumulation and heavy dependence on subsistence activities. Thus, the measurement of purchasing power through family income may not be the most appropriate way to measure access to food in this type of population.^30^ It is known, however, that stunting is predominantly associated to families' economic status. Thus, the positive association found here between low income and children's stunting refers to the premise that the increase in families' income is directly related to nutritional status, as a higher income enables greater access to food.^28^ The data shown here corroborate this premise, as the higher mean *z* scores were identified among children belonging to the higher-income families, when compared with those whose income was below the used cutoff point.

The sensitive association between the presence of iron-deficiency anemia and delayed growth in children has been reported in scientific literature. However, there was no statistically significant association between stunting and anemia in this group, despite the high prevalence rates found for the two diseases. In fact, malnourished children often have anemia, among other associated nutritional deficiencies, and it is considered that malnutrition is characterized by the simultaneous deficiency of several nutrients.^31^ Considering the high prevalence of delayed growth (13.4%) and the simultaneous occurrence of iron deficiency anemia (57.7%) in this population group, it is recommended that the investigation of these diseases and their associated factors be more thorough. About this aspect, it is noteworthy that, when analyzing the variables associated with nutritional status in this group, we did not intend to establish a cause-and-effect association. This study carried out an assessment aiming to detect more or less nutritional vulnerability in a still little-investigated population group.

It can be presupposed, as an immediate cause level, that the inadequate birth weight may prevent children from this population from reaching their full linear growth potential.^32^ The statistical association between malnutrition and low birth weight found here, may result from factors related to pregnancy that were not investigated here.^33^ The established hypothesis is that a poor nutritional status at this stage of life is an important and significant risk factor for the subsequent occurrence of delayed growth. That is what the data shown here seem to confirm, identifying lower mean *z* scores in children with low birth weight,^32^ a fact also confirmed by Kühl et al.,^26^ who found an association between delayed growth and low birth weight in *Kaingang* children.

Regarding the comparison of the growth curves of the studied population with the WHO reference curves, it can be clearly observed that the children of the *karapotó* people living in Plak-Ô reservation and Terra Nova settlement have lower *z* score values than the used cutoff point. The WHO curves constitute a set of data that represents the best description of physiological linear growth of healthy children under favorable environmental conditions, regardless of their ethnic background. It is also noteworthy that an important feature of these curves is to represent an international and multiethnic sample used to assess indigenous populations.^1^^-^ 3 ^,^ 5 ^,^ 26 ^,^ 27

Thus, the slopes displaced to the left found in the three curves in relation to the used reference standard, according to place of residence, show lower mean z scores than expected in a healthy population. That is, the slopes of the curves shown here define delayed growth observed in the children of the assessed population.

A limitation of the study is its cross-sectional design, an aspect that prevents cause-and-effect inferences. Therefore, the findings should be treated with caution until a longitudinal assessment of risk factors is carried out. Although cross-sectional studies do not allow causality inference, they are essential to establish hypotheses and direct the planning of prospective studies to establish clear associations between the conditioning/determinant factors of children's stunting. Despite the aforementioned limitations, the study is unprecedented for this people, represents the universe of *karapotó* children and shows that malnutrition is still present as an important nutritional disorder in this population.

It is important to emphasize that malnutrition in children has a multiple determination of a complex network of factors, involving biological, demographic, socioeconomic and cultural aspects, among others.^5^^,^ 6 ^,^ 8 ^,^ 27 ^,^ 34 Thus, the child, especially those from traditional people, cannot be seen as a single unit. To explain the dynamism and diversity of the nutritional status of Brazilian indigenous populations demand an interaction of anthropological, biological and social factors, considering children in their context. In this population, the observed prevalence of delayed growth was significant when compared to the non-indigenous population in the country, divergent from the references established by the WHO. Among the associated factors, the unfavorable conditions of maternal literacy, family income and low birth weight are emphasized.

Thus, given the known sensitivity of these factors to express a population's life standard, it is crucial to consider the specifics of the assessed group, in order to implement appropriate strategies to solve the identified problems. Therefore, it is considered essential to establish policies that take into account the culture and eating habits and respect traditional knowledge and biodiversity of this people.
